# First Report on Association of Hyperuricemia with Type 2 Diabetes in a Vietnamese Population

**DOI:** 10.1155/2019/5275071

**Published:** 2019-09-02

**Authors:** Tran Quang Binh, Pham Tran Phuong, Nguyen Thanh Chung, Bui Thi Nhung, Do Dinh Tung, Tran Quang Thuyen, Duong Tuan Linh, Bui Thi Thuy Nga, Nguyen Anh Ngoc, Le Danh Tuyen

**Affiliations:** ^1^National Institute of Nutrition, 48B Tang Bat Ho Street, Hanoi, Vietnam; ^2^National Institute of Hygiene and Epidemiology, 1 Yersin, Hanoi, Vietnam; ^3^Dinh Tien Hoang Institute of Medicine, 20 Cat Linh, Dong Da, Hanoi, Vietnam; ^4^National Institute of Diabetes and Metabolic Disorders, 1 Ton That Tung, Hanoi, Vietnam; ^5^Vietnam Military Medical University, 160 Phung Hung Street, Ha Dong, Hanoi, Vietnam

## Abstract

**Background:**

Uric acid is a powerful free-radical scavenger in humans, but hyperuricemia may induce insulin resistance and beta-cell dysfunction. The study aimed to evaluate the association between hyperuricemia and hyperglycemia, considering the confounding factors in a Vietnamese population.

**Methods:**

A population-based cross-sectional study recruited 1542 adults aged 50 to 70 years to collect data on socioeconomic status, lifestyle factors, and clinical patterns. Associations between hyperuricemia and hyperglycemia (isolated impaired fasting glucose (IFG), isolated impaired glucose tolerance (IGT), combined IFG-IGT, and type 2 diabetes (T2D)) were evaluated by multinomial logistic regression analysis in several models, adjusting for the confounding factors including socioeconomic status, lifestyle factors, and clinical measures.

**Results:**

Uric acid values were much higher in IFG, IFG-IGT, and T2D groups compared to those in the normal glucose tolerance (NGT) group. The significant association of hyperuricemia with IFG, IFG-IGT, and T2D was found in the model unadjusted and remained consistently in several models adjusted for socioeconomic status, lifestyle factors, and clinical patterns. In the final model, the consistent hyperglycemia risk was found in total sample (OR = 2.23 for IFG, OR = 2.29 for IFG-IGT, and 1.75 for T2D, *P* ≤ 0.006) and in women (OR = 2.90 for IFG, OR = 3.96 for IFG-IGT, and OR = 2.49 for T2D, *P* < 0.001) but not in men.

**Conclusions:**

It is the first report in Vietnamese population suggesting the significant association of hyperuricemia with IFG, IFG-IGT, and T2D; and the predominant association was found in women than in men, taken into account the confounding factors.

## 1. Introduction

Uric acid is produced during the exogenous metabolic breakdown of purines from dietary intake, and it is also a product from the endogenous degradation from dead cells. Most serum uric acid (SUA) is freely filtered, and approximately 90% of filtered uric acid is reabsorbed, showing its essential role in human body [1]. Uric acid plays a considerable physiological role as strong reactive oxygen species, a powerful free radical and peroxynitrite scavenger [2, 3] and a major plasma antioxidant [4]. However, there has been also a controversial opinion about prooxidative and antioxidant properties of uric acid [5]. Uric acid represents a marker for high levels of damaging oxidative stress [6]. Moreover, hyperuricemia induces insulin resistance [7], while hyperinsulinemia caused by insulin resistance increases SUA concentration by both reducing renal uric acid secretion [8] and accumulating substrates for uric acid production [9]. The relation between elevated SUA and type 2 diabetes remains controversial. Elevated SUA was an independent risk factor for type 2 diabetes in some studies in Sweden [10], China [11], Netherlands [12], and Brazil [13]. However, the inverse association with diabetes was found in several reports. SUA levels tended to increase with increasing fasting plasma glucose levels in nondiabetic individuals but decrease in diabetic individuals in a general Chinese population [14]. Higher SUA levels were inversely associated with type 2 diabetes in a representative sample of adults in United States [15]. Furthermore, in Japanese men, uric acid was negatively associated with diabetes in a cross-sectional study [16], while prospective studies reported inconsistent findings that SUA level was not associated [17] or associated [18] with an increased risk for type 2 diabetes. The inconsistent association between elevated SUA and type 2 diabetes may be explained by confounding factors including socioeconomic status, lifestyle-related factors, and clinical measures (body mass index, blood pressures, and dyslipidemia). Recently, a recommendation that the correlation between SUA and type 2 diabetes requires further evaluation has been noted from a systematic review and meta-analysis including 970 studies in 61,714 participants [19]. Therefore, we conducted a population-based cross-sectional study to investigate the association between hyperuricemia and hyperglycemia including impaired fasting glucose, impaired glucose tolerance, and type 2 diabetes, considering the confounding factors in a Vietnamese population. The gender difference in the association was also reported.

## 2. Methods

### 2.1. Setting and Study Subjects

The cross-sectional study was a part of the DiaMetS-VN population-based prospective study designed to conduct in Ha Nam province, Vietnam, from July to December 2016. Ha Nam province locates in the southwest of the Red River Delta and 50 km far from Hanoi City. It has a population of about 800,000 inhabitants living in 108 rural communes and 6 urban wards [20]. The Ethics Committee of the National Institute of Hygiene and Epidemiology, Vietnam, approved the protocol of the survey (IRB-VN01057-34/2016). All the participants belonged to the Viet ethnic group, and they provided written informed consent before taking part in the study.

### 2.2. Serum Biochemical Analysis

Serum uric acid was measured by the uricase-based methods using a fully automatic biochemistry analyzer (FACA-401 autoanalyzer, Labomed Inc., USA) with a commercial kit (Erbra, Germany). In the procedure, uric acid is converted by uricase to allantoin and hydrogen peroxide, and the amount of hydrogen peroxide produced is measured. Hyperuricemia was classified as follows: for women, uric acid level ≥360 *μ*mol/L (6 mg/dL) and for men ≥420 *μ*mol/L (7.0 mg/dL) [21].

Serum glucose and lipid profile were quantified using laboratory methods as reported previously [22]. Dyslipidemia [23] is defined as low-density lipoprotein cholesterol level <40 mg/dL for men and <50 mg/dL for women, and total cholesterol, low-density lipoprotein cholesterol, and triglyceride levels ≥200, ≥130, and ≥130 mg/dL, respectively. The glycemic status of subjects was determined using fasting plasma glucose (FPG) and 2h plasma glucose (2h-PG) by the oral glucose tolerance test with 75 g glucose. The glycemic status was classified as normal glucose tolerance (NGT) when FPG < 5.6 mmol/L and 2h-PG < 7.8 mmol/L, as isolate impaired fasting glucose (IFG) when 5.6 ≤ FPG ≤ 6.9 mmol/L and 2h-PG < 7.8 mmol/L, as isolated impaired glucose tolerance (IGT) when FPG < 5.6 mmol/L and 7.8 ≤ 2h-PG ≤ 11.0 mmol/L, as combined IFG and IGT (IFG−IGT) when 5.6 ≤ FPG ≤ 6.9 mmol/L and 7.8 ≤ 2h-PG ≤ 11.0 mmol/L, and as type 2 diabetes (T2D) when FPG ≥ 7.0 mmol/L and/or 2h-PG ≥ 11.1 mmol/L or previous diagnosis of diabetes and current use of drug for its treatment, according to guidelines of the World Health Organization and International Diabetes Federation [24].

### 2.3. Socioeconomic, Lifestyle, and Clinical Covariates

All participants were directly interviewed by trained surveyors to complete a structured questionnaire as presented previously [22]. Socioeconomic variables included age, gender, residence, marital status, education level (elementary, intermediate, secondary, and postsecondary), occupation, and income level. Lifelong occupation was defined as the occupation that the subject engaged in most frequently in the life. It was categorized as heavy occupation (farmer and manual worker) and nonheavy occupation (office clerks, teacher, retired worker, and houseworker).

Lifestyle factors were composed of consumption of wine and beer (none, <1 drink/mo, ≥1 drink/mo to <1 drink/wk, 1 drink/wk to ≤ 1 drink/d, and ≥2 drink/d, one drink was defined as a 50 ml cup of rice wine at about 30%), smoking (never, current, and former), consumption of sugary drinks (<1 drink/mo, ≥1 drink/mo to <1 drink/wk, 1 drink/wk to <1 drink/d, and ≥1 drink/d, one drink was defined as a 330 ml cup), sporting habit (none, light, heavy), time spent for night's sleep (7 h, ≤6 h, and ≥8 h), siesta (per 30 min), watching television (3 h and >3 h), and leisure sitting (4 h and >4 h).

Clinical patterns were characterized by body mass index (BMI), systolic blood pressure, diastolic blood pressure, and dyslipidemia. Body mass index was calculated as weight per square of height (kg/m^2^).

### 2.4. Statistical Analysis

Quantitative variables were checked for normal distribution and compared using One-Way ANOVA or independent-sample *T* test. Kruskal–Wallis or Mann–Whitney *U* test was used to compare quantitative variables without normal distribution. Frequencies of category variables were compared by Pearson's chi-squared test.

Multinomial logistic regression analysis was performed to test several models for the associations between hyperuricemia and hyperglycemia, adjusting for confounding factors including (i) socioeconomic status: age, sex, residence, education level, occupation, marital status, and income level; (ii) lifestyle-related factors: consumption of wine and beer, smoking, consumption of sugary drinks, sporting habit, time spent for night's sleep, siesta, watching television, and leisure sitting; and (iii) clinical measures: BMI, systolic blood pressure, diastolic blood pressure, and dyslipidemia. Data are expressed as odds ratios with 95 percent confidence intervals (CI). Associations were considered statistically significant at two-sided *P* values of less than 0.05 for all the analyses. The above statistical procedures were performed using SPSS version 16.0.

## 3. Results

### 3.1. Characteristics of the Study Cohort

Table 1 shows the characteristics of the subjects according to blood glucose levels. Mean (±SD) of age and BMI of the subjects were 56.5 (±6.7) years and 21.9 (±2.7) kg/m^2^, respectively. The diabetes group had significant higher age, weight, BMI, and blood pressures than the NGT group. There was no significant difference of weight, height, and BMI among NGT, IFG, IGT, and IFG-IGT groups.

### 3.2. Uric Acid Concentrations by Gender and Glucose Levels

Table 2 shows the comparison of SUA concentration among 5 glucose levels (NGT, IFG, IGT, IFG-IGT, and diabetes) in men, women, and total sample. SUA values were much higher in IFG, IFG-IGT, and diabetes groups compared to the NGT group. There was no significant difference of SUA value between NGT and IGT groups. Men had higher SUA concentration than women in NGT, IFG, and diabetes groups.

### 3.3. Association between Elevated Uric Acid and Hyperglycemia

Table 3 shows the association of hyperuricemia with hyperglycemia including IFG, IGT, IFG-IGT, and T2D in several models considering the influence of socioeconomic conditions (age, sex, residence, education level, occupation, marital status, and income level) in the Model 2, adding lifestyle-related factors (consumption of wine and beer, smoking, consumption of sugary drinks, sporting habit, time spending for night's sleep, siesta, watching TV, and leisure sitting) in the Model 3, and further clinical measures (BMI, blood pressures, and dyslipidemia) in the Model 4. The significant association of hyperuricemia with IFG, IFG-IGT, and diabetes was found in the Model 1 unadjusted and remained consistently in several models adjusted for socioeconomic status (Model 2), lifestyle-related factors (Model 3), and clinical patterns (Model 4). In the final Model 4, after adjustments for socioeconomic status, lifestyle factors, and clinical pattern, the hyperuricemia was found to be an independent risk factor for hyperglycemia in the total sample (OR = 2.23 for IFG, OR = 2.29 for IFG-IGT, and 1.75 for T2D, *P* ≤ 0.006) and in women (OR = 2.90 for IFG, OR = 3.96 for IFG-IGT, and OR = 2.49 for T2D, *P* < 0.001). Such an association was not observed for IGT. When analyzing by stratum of gender, the associations remained significantly in females, but not in males.

**Figure 1:**
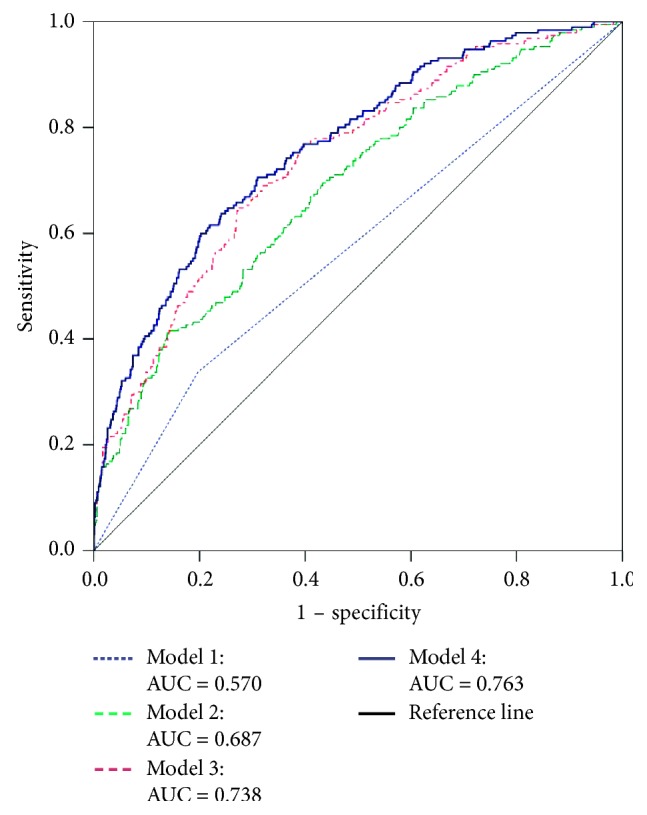
Serum uric acid receiver-operating characteristic curve for type 2 diabetes. Model 1: unadjusted. Model 2: adjusted for socioeconomic status (age, gender, residence, marital status, education level, occupation, and income level). Model 3: Model 2 adjusted for lifestyle factors (consumption of wine and beer, smoking, consumption of sugary drinks, sporting habit, time spending for night's sleep, siesta, watching TV, and leisure sitting). Model 4: Model 3 adjusted for clinical patterns (body mass index, systolic blood pressure, diastolic blood pressure, and dyslipidemia).

Adjusting for covariates improved the area under ROC curve significantly (Figure 1) from 0.570 (*P*=0.002) in the unadjusted model to 0.687, 0.738, and 0.763 (*P* < 0.001), respectively, in models adjusted for socioeconomic status (Model 2), lifestyle factors (Model 3), and clinical patterns (Model 4). Adding the hyperuricemia variable in the final Model 4 improved the area under the receiver-operating characteristic curve slightly (from 0.758 to 0.763, *P* < 0.001).

## 4. Discussion

In this study, we have found the significant association of hyperuricemia acid with IFG, IFG-IGT, and diabetes, independent of the confounding factors. Given the multifactorial pattern of type 2 diabetes, the association of SUA with type 2 diabetes varies among populations depending on the socioeconomic status, lifestyle factor, genetic background, and clinical risk factor profile of each population [25]. Our finding is consistent with several reports [10–13]. The inconsistent findings on the association between SUA and T2D even in one population, i.e., Japanese men [16–18], may be resulted from the confounding factors which are not considered in the analysis of the association. Our analysis considered the important T2D risk factors including gender, age, alcohol consumption, smoking, consumption of sugary drinks, BMI, blood pressures, and lipid profile as well as other socioeconomic and lifestyle-related factors.

In line with a report in Japan [18], the present study showed the association of SUA with IFG and T2D in Vietnamese population with the BMI mean <25 (21.9 ± 2.7) kg/m^2^, whereas the other reported that the association between SUA and incident prediabetes was not significant among Caucasian population with BMI <25 kg/m^2^ [26]. This indicates the important difference in BMI between Vietnamese and Caucasians in the pathogenesis of type 2 diabetes [27], and Vietnamese may develop type 2 diabetes with smaller increases in BMI than Taiwanese [28].

The significant difference of association by gender was found in our cohort. In agreement with the study in German population [29], the present study reported the significant association of hyperuricemia with hyperglycemia risk in females (OR = 2.90 for IFG, OR = 3.96 for IFG-IGT, and OR = 2.49 for T2D, *P* < 0.001) but not in males. In addition, the association was found in the IFG group but not in the IGT group. These findings may be explained by that SUA affects women more strongly in the early stages of glucose intolerance development, whereas it affects men more strongly in more advanced stages [26]. Previous studies supported the similar finding that SUA may be a useful predictor of type 2 diabetes in older adults with impaired fasting glucose [13] and a strong positive association between SUA and incident prediabetes in females rather than in males [30].

Both insulin resistance and *β*-cell dysfunction play determinate roles in the pathogenesis of type 2 diabetes. High SUA directly induces insulin resistance by impairing glucose tolerance and inhibiting insulin signaling in vivo and by inducing oxidative stress in vitro [31]. Higher SUA is associated with greater insulin secretion ability at the early stage of the disease, but it seems to reduce residual *β*-cell function more rapidly [32]. Increased SUA has a direct negative effect on *β*-cell function, which could cause *β*-cell death and dysfunction by activation of the NF-*κ*B and iNOS-NO signal axis [33]. Collectively, elevated SUA has a direct effect on both insulin resistance and *β*-cell dysfunction in the pathogenesis of type 2 diabetes. These evidences support the significant association of hyperuricemia with IFG, IFG-IGT, and diabetes in the Vietnamese population.

There has been an inconsistent relation between hyperuricemia and IGT. In agreement with other reports [29], the present study showed no association between hyperuricemia and IGT status, whereas SUA was related to IGT in the Chinese adults, independent of other conventional metabolic risk factors [34]. Based on the oral glucose tolerance test, the previous study indicated that IFG was due to impaired basal insulin secretion and preferential resistance of glucose production to suppression by insulin (as reflected by fasting hyperglycemia despite normal plasma insulin concentrations and increased HOMA-IR), whereas IGT mainly resulted from reduced second-phase insulin release and peripheral insulin resistance (as reflected by reduced clamp-determined insulin sensitivity) [35]. The aetiologies of IFG and IGT also seem to differ, with IFG being predominantly related to genetic factors, smoking, and male sex, whereas IGT is predominantly related to physical inactivity, unhealthy diet, and short stature [36]. Both IFG and IGT had inappropriately elevated glucagon secretion. Subjects with IFG had predominant reduced hepatic insulin sensitivity and normal skeletal muscle insulin sensitivity, while subjects with IGT had near-normal hepatic and moderate to severe skeletal muscle insulin resistance [37]. IFG induced dysfunction and/or chronic low mass of beta cell and altered glucagon-like peptide-1 secretion, whereas IGT induced progressive loss of beta-cell function and reduced secretion of glucose-dependent insulinotropic polypeptide. It is necessary to conduct further studies to elucidate the relation between uric acid concentrations and IGT status.

Using medications with potential effect on SUA levels may be the confounding factor for the association between hyperuricemia and hyperglycemia. This study design as a population-based study can reduce the confounding factor of drugs, which usually happens in a hospital-based study. In the total of 1542 participants, there were 46 (3%) patients with previously diagnosed diabetes. Only 15 patients recalled the antidiabetic drugs: insulin (*n* = 8), metformin (*n* = 6), and gliclazide (*n* = 1). Among antidiabetic drugs, SGLT2 inhibitors have a potential effect of reducing SUA levels. In the study conducted in 2016, SGLT2 inhibitors were not recommended for patients with diabetes in Ha Nam province because these drugs were costly and not paid by Vietnam Health Insurance. Therefore, using antidiabetic drugs may not be the confounding factor for the observed association.

The present findings must be interpreted in the context of several limitations. First, the findings obtained from a cross-sectional study cannot give any causative relation. Next, insulin resistance and beta-cell function were not evaluated, limiting the analysis of the most important pathogenesis of type 2 diabetes and other hyperglycemic status. Third, the study did not clarify antihypertensive drugs as confounding factors in 128 (8.3%) previously diagnosed patients with hypertension. Lastly, genetic factors were not included in the analysis, so the findings could not explained by genetic variances in the population.

## 5. Conclusions

In summary, the study showed that there was a significant association of hyperuricemia with IFG, IFG-IGT, and diabetes in the Vietnamese population, and the predominant association was found in females than in males, taken into account the confounding factors.

## Figures and Tables

**Table 1:** Characteristics of the studied subjects according to blood glucose levels.

Variables	NGT (*n* = 998)	IFG (*n* = 176)	IGT (*n* = 121)	IFG + IGT (*n* = 57)	Diabetes (*n* = 190)	*P* value
Gender (male)	318 (31.9)	54 (30.7)	49 (40.5)^*∗*^^#^	24 (42.1)^*∗*^^#^	77 (40.5)^*∗*^^#^	0.033
Age (year)	56.0 ± 6.7	56.2 ± 6.6	57.5 ± 6.6	58.3 ± 6.1^*∗*^^#^	58.3 ± 6.7^*∗*^^#^	<0.001
Height (cm)	155.9 ± 7.3	156.2 ± 7.3	156.3 ± 6.9	156.0 ± 7.2	156.5 ± 7.7	0.825
Weight (kg)	52.9 ± 8.1	54.0 ± 8.4	54.1 ± 7.9	54.9 ± 9.1	55.4 ± 8.4^*∗*^	0.001
BMI (kg/m^2^)	21.8 ± 2.6	22.1 ± 2.6	22.1 ± 2.9	22.5 ± 3.0	22.6 ± 2.8^*∗*^	0.001
SBP (mmHg)	122.5 (112.5–137.7)	126.8^*∗*^ (114.5–137.5)	129.0^*∗*^^#^ (117.3–143.8)	139.0^*∗*^^#^ (120.8–149.5)	134.5^*∗*^^#^ (121.0–147.1)	<0.001
DBP (mmHg)	80.0 (71.0–87.5)	80.0 (70.0–84.8)	82.5^*∗*^^#^ (75.0–91.3)	85.5^*∗*^^#^ (75.3–92.3)	82.0^*∗*^^#^ (73.9–90.0)	<0.001

Data are expressed as the mean with standard deviation and median (interquartile range), except for gender as number (percentage). *P* value for difference between the groups was calculated from the one-way ANOVA or Kruskal–Wallis test or chi-squared test: ^*∗*^vs. NGT group and ^#^vs. IFG group. NGT, normal glucose tolerance; IFG, impaired fasting glucose; IGT, impaired glucose tolerance; BMI, body mass index; SBP, systolic blood pressure; DBP, diastolic blood pressure.

**Table 2:** Uric acid concentration among glucose levels of subjects.

Glucose level	Total		Men		Women		*P*_1_ value
	*n*	Median (interquartile range)	*n*	Median (interquartile range)	*n*	Median (interquartile range)	
NGT	998	294.3 (241.6–357.9)	319	331.8 (265.0–410.7)	679	276.7 (230.8–337.7)^*ϕ*^	<0.001
IFG	176	358.7 (244.6–406.1)^*∗*^	54	391.2 (343.1–454.5)^*∗*^	122	337.3 (225.2–392.0)^*∗*^^*ϕ*^	<0.001
IGT	121	295.6 (236.8–379.9)^#^	49	315.3 (240.5–420.9)^#^	72	285.5 (215.1–354.7)^#^	0.073
IFG and IGT	57	370.4 (279.3–418.0)^*∗*†^	24	389.5 (331.0–425.8)^*∗*†^	33	359.7 (256.7–410.0)^*∗*†^	0.056
Diabetes	190	343.9 (252.3–410.0)^*∗*†^	77	371.7 (307.9–426.7)^*∗*^	113	304.4 (238.5–398.0)^*∗*^^*ϕ*^	0.001
*P*_2_ value		<0.001		≤0.001			<0.001

*P*_1_ value for difference between men and women was calculated from the Mann–Whitney test: ^*ϕ*^vs. men. *P*_2_ value for difference among glucose levels (NGT, IFG, IGT, IFG-IGT, and diabetes) in men, women, and total sample was calculated from the Kruskal–Wallis test: ^*∗*^vs. NGT group, ^#^vs. IFG group, and ^†^vs. IGT group. NGT, normal glucose tolerance; IFG, impaired fasting glucose; IGT, impaired glucose tolerance.

**Table 3:** Association of elevated serum uric acid with hyperglycemia in multinomial logistic regression adjusted for confounding factors.

Model	IFG		IGT		IFG-IGT		Diabetes	
	OR (95% CI)	*P* value	OR (95% CI)	*P* value	OR (95% CI)	*P* value	OR (95% CI)	*P* value
Total (*n* = 1542)								
Model 1	2.58 (1.833.62)	<0.001	1.18 (0.75–1.85)	0.487	2.57 (1.48–4.48)	0.001	2.08 (1.48–2.92)	<0.001
Model 2	2.33 (1.63.3.32)	<0.001	1.15 (0.73–1.83)	0.544	2.50 (1.42–4.38)	0.001	1.96 (1.38–2.78)	<0.001
Model 3	2.31 (1.61–3.32)	<0.001	1.12 (0.71–1.79)	0.624	2.76 (1.55–4.90)	0.001	1.99 (1.39–2.83)	<0.001
Model 4	2.23 (1.54–3.23)	<0.001	1.08 (0.67–1.74)	0.744	2.29 (1.27–4.11)	0.006	1.75 (1.22–2.52)	0.002

Women (*n* = 1019)								
Model 1	3.27 (2.17–4.93)	<0.001	1.14 (0.61–2.10)	0.685	4.43 (2.18–9.02)	<0.001	2.78 (1.81–4.28)	<0.001
Model 2	2.72 (1.77–4.19)	<0.001	1.14 (0.62–2.13)	0.672	3.91 (1.87–8.16)	<0.001	2.57 (1.65–4.02)	<0.001
Model 3	2.87 (1.84–4.50)	<0.001	1.21 (0.64–2.27)	0.559	4.44 (2.08–9.47)	<0.001	2.79 (1.76–4.41)	<0.001
Model 4	2.90 (1.83–4.58)	<0.001	1.07 (0.56–2.03)	0.837	3.96 (1.83–8.54)	<0.001	2.49 (1.55–3.99)	<0.001

Men (*n* = 523)								
Model 1	1.57 (0.84–2.93)	0.154	1.14 (0.57–2.25)	0.717	1.05 (0.40–2.73)	0.924	1.26 (0.72–2.19)	0.421
Model 2	1.85 (0.96–3.55)	0.065	1.14 (0.57–2.28)	0.712	1.05 (0.40–2.79)	0.917	1.36 (0.77–2.41)	0.297
Model 3	1.72 (0.88–3.36)	0.114	1.06 (0.52–2.16)	0.879	1.10 (0.40–3.04)	0.859	1.36 (0.76–2.45)	0.304
Model 4	1.55 (0.78–3.08)	0.207	1.10 (0.53–2.29)	0.808	0.79 (0.27–2.29)	0.667	1.19 (0.65–2.19)	0.576

Model 1: unadjusted. Model 2: adjusted for socioeconomic status (age, gender, residence, marital status, education level, occupation, and income level). Model 3: Model 2 adjusted for lifestyle factors (consumption of wine and beer, smoking, consumption of sugary drinks, sporting habit, time spent for night's sleep, siesta, watching television, and leisure sitting). Model 4: Model 3 adjusted for clinical patterns (body mass index, systolic blood pressure, diastolic blood pressure, and dyslipidemia). IFG, impaired fasting glucose; IGT, impaired glucose tolerance.

## Data Availability

The data used to support the findings of this study are available from the corresponding author upon request.

## Abbreviations

BMI: Body mass index
NGT: Normal glucose tolerance
IFG: Impaired fasting glucose
IGT: Impaired glucose tolerance
FPG: Fasting plasma glucose level
OGTT: Oral glucose tolerance test
SBP: Systolic blood pressure
DBP: Diastolic blood pressure
SUA: Serum uric acid.
